# The informational content of subjective expectations for health service use

**DOI:** 10.1186/s12913-021-06464-7

**Published:** 2021-05-17

**Authors:** Nathan Kettlewell

**Affiliations:** University of Technology Sydney, 15 Broadway, Ultimo NSW, 2007 Australia

**Keywords:** Subjective expectations, Beliefs, Subjective probabilities, Health insurance, Healthcare demand, D82, D84, I11, I12, I13

## Abstract

**Background:**

This study aims to evaluate the informational content of people’s subjective probability expectations for using various health services.

**Methods:**

Using a sample of 1,528 Australian adults (25-64 years), I compared stated probabilities of visiting various health service providers (hospitals, dentists, optometrists, physiotherapists and related care providers, naturopaths and massage therapists) with past utilization and with predicted utilization estimated out-of-sample. I also estimated whether past utilization and subjective expectations were predicted by the same covariates. Finally, I estimated whether subjective expectations had predictive power for the choice to purchase private health insurance conditional on past utilization and other controls.

**Results:**

Subjective expectations closely reflect patterns of observed utilization, are predicted by the same covariates as observed utilization, and correlate with objective measures of risk. Subjective expectations also add predictive power to models estimating insurance take-up, even after conditioning on prior health care use and other risk factors.

**Conclusion:**

The findings are indicative that on average people form quite accurate expectations, and support collecting subjective expectations about health services in household surveys for use in applied research.

**Supplementary Information:**

The online version contains supplementary material available at (10.1186/s12913-021-06464-7).

## Introduction

Modelling demand for health services is challenging since observable risk factors often provide limited information on individual risk. Further, observable risk factors do not necessarily capture people’s risk perceptions. If beliefs about healthcare use are biased, then unadjusted correlations between objective risk factors and behaviors, such as insurance purchase, may be weaker than if perceptions were controlled for, leading to incorrect inferences about people’s behavior.

In many fields, researchers have used subjective probability expectations as a way of dealing with the unobservability of beliefs. Examples include job insecurity [1], future income [2, 3], long term care [4] and investment markets [5, 6]. Studies in the health domain have generally focussed on subjective expectations for specific diseases, adverse health and mortality. They have tackled diverse questions, for example, how beliefs about HIV risk are shaped by information [7] and affect sexual behavior [8], how people self-select into annuity insurance [9], how expectations about risks from smoking [10–12] and alcohol consumption [13, 14] inform behavior and how expectations towards afflictions like influenza, breast cancer and heart disease affect preventative care use [15]. These studies demonstrate the wide-ranging application of subjective expectations to health behaviors research.

In this paper I focus on subjective expectations for health service use, rather than expectations for particular health outcomes. This distinction is important; in many situations, final demand for health services is of primary interest, rather than the foundations of that demand. For example, policy makers and insurers are interested in people’s expected probability of hospital admission, in part because this is expected to be the most fundamental driver of demand for private insurance. This is particularly true in mixed public/private healthcare systems where consumers are protected from the intensive margin because they either face only the cost of the deductible when they receive private treatment, or can access free public care. Accurate predictions of health service use are necessary for effective resource management by those who require these data, such as hospital managers and departments of health. At the same time, if we learn that people are bad at predicting their health service use, or predict use in a biased way, this may motivate efforts to improve the quality of people’s beliefs so they can make more informed health related decisions. Finally, it is notable that any effort to predict health service use from expectations around health conditions ignores the other drivers of health care use (e.g. income, insurance coverage). Further, eliciting a single probability over the likelihood of (say) hospital admission is also likely to generate more accurate information than eliciting a high dimensional vector of probabilities over all the possible diseases and risk factors that could potentially lead to a hospital admission (and is certainly more feasible). It therefore offers a practical way forward when coarsely defined service use is the variable of interest.

The primary goal of this paper is to assess whether subjective expectations are indicative of actual risk in the case of health service use, specifically hospitalizations and visits to ancillary care providers (dentists, optometrists, physiotherapists and related care providers, naturopaths and massage therapists). While subjective expectations have proved to be reliable predictors of objective risk and behavior in a number of settings (see [16, 17] for reviews), they have not yet been assessed for health services^1^. Further, there is reason to question how much these measures reflect actual probabilities in the health care domain. In a classic study on biased beliefs, [19] provide evidence of systematic bias in judgements of risk of death from various illnesses and events. Overall, people tend to overweight (underweight) low (high) probability events. [20, 21] show that people are systematically overconfident with respect to their risk of developing health problems (see also [22]). At the same time, people overestimate their risk of death from influenza, developing breast and lung cancer and suffering from heart disease and stroke when compared to objective predictions [12, 15]. [23] find that while many people have accurate predictions, on average people over-predict their risk of relatively infrequent conditions like diabetes, stroke, heart attack and lung disease, but under-predict risk of hypertension, which is more common. People also over-predict risks associated with smoking [10, 11] and alcohol [13, 14]. These findings suggest that people may have biased perceptions about health service use as well.

On the other hand, it may be easier for people to form unbiased expectations about health service use than disease risk. People will often have personal experience with health service providers to draw on. They can think about how frequently they, or their friends and family, have been hospitalized in the past. Some service use will also be planned in advance. Finally, frequencies for these events are generally much higher than the risk of any particular ailment, which may suppress the tendency for people to overweight low probability events.

I elicit subjective expectations in a large online survey, conducted in Australia, where people are asked to state their likelihood (0-100%) of utilizing various types of health services. I assess the informational content of people’s responses in several ways. I find that these measures are positively correlated with objectively predicted risk, that they closely match the actual rates of health service use, and that the partial correlations between covariates like age and gender and expected vs. realised outcomes are similar. Bias in average expectations is generally in the direction of underestimating future health service use. To further explore the informational value in these data, I test whether subjective expectations predict demand for private health insurance and find that expectations independently predict insurance for services excluded from the public safety net, even after conditioning on prior health service use and observable risk factors.

This research has implications for how we think about choice frictions in health care decision making. The results are indicative that expectations are (on average) fairly accurate, implying there may be little need to correct for biased beliefs. The results also highlight the research-value in collecting subjective expectations about future health service use. Questions on expectations could be included in large household surveys at minimal cost.

The paper is organized as follows. “Data” section describes the data, “Assessing subjective expectations” section explores the extent to which subjective expectations concord with actual risk, “Using subjective expectations to predict insurance take-up” section tests the predictive power of expectations and “Conclusion” section concludes.

## Data

### Datasets

This study uses two survey datasets. The primary dataset (Online Survey) is a sample of 1,528 Australians aged 25-64 years who were surveyed between 10-21 December 2015^2^. These people were recruited by the market research company Qualtrics from their online research panel. The main component of the survey was a discrete choice experiment related to insurance choice, which has been analysed elsewhere [24]. In addition to this experiment, respondents were asked a number of questions about demographics, risk preferences and subjective expectations regarding health service use. Quotas for age, sex and education were used to improve representativeness of the sample. Table 1 compares the Online Survey sample to population benchmarks and shows that this sample is observationally similar to the general Australian population of 25-64 year olds on many dimensions, although does have lower income and employment and is somewhat older.

**Table 1:** Descriptive statistics (mean values)

	Sample	Population	Difference
Age 25-34	0.228	0.272	-0.044^***^
Age 35-44	0.234	0.254	-0.020^*^
Age 45-54	0.227	0.251	-0.024^*^
Age 55-64	0.311	0.223	0.088^***^
Male	0.492	0.491	0.001
University	0.265	0.283	-0.018
Couple	0.619	0.613	0.006
Employed	0.616	0.703	-0.087^***^
PHI (hospital)	0.491	0.497	-0.006
PHI (ancillaries)	0.501	0.548	-0.047^***^
*Household income*			
<<DOLLAR/>60K	0.435	0.239	0.196^***^
<DOLLAR/>60K– <<DOLLAR/>125K	0.392	0.373	0.019
<DOLLAR/>125K+	0.173	0.388	-0.215^***^

Note: Sample size is 1,528. Couple refers to those in either registered marriages or de-facto relationships. University indicates highest qualification is bachelors degree or higher. Population values for age, sex, university, couple and employed are from the 2016 Australian Bureau of Statistics (ABS) Census. APRA figures (June 2016) and the 2016 ABS Census have been used for PHI coverage. Population figures for income are weighted values from the 2015 wave of HILDA. t-tests for whether the sample mean equals the population mean treat the population mean as known. ^*^ *p*<0.10, ^**^ *p*<0.05, ^***^ *p*<0.01

The second dataset is the Household, Income and Labour Dynamics in Australia Survey (HILDA). HILDA is a nationally representative household panel that has been tracking Australian households since 2001. It began with a sample of 19,914 individuals belonging to 7,682 households. In 2011 the sample was topped-up with an additional 2,153 households. I use data from the 2013 wave of HILDA, which is the most temporally close wave to the Online Survey (2015) in which information on private health insurance and health service use is available (excluding waves after 2015). Data from HILDA are used to build prediction models for health service use which are then applied to the Online Survey sample in order to assess how closely subjective expectations correlated with predicted risk. There are around 9,000 individuals used in the prediction models (with sample sizes varying slightly by outcome variable) after restricting the HILDA sample to those aged 25-64 years with no missing information on the covariates common to both surveys.

### Institutional background

Before discussing the main variables, a brief discussion about health care in Australia is necessary. All Australians have access to free public insurance for hospitalization through a scheme known as Medicare, which covers admissions to public hospitals. People can also purchase private hospital insurance to cover fees at private hospitals (or as a private patient in a public hospital), often with co-payments. Public hospitals are a high quality alternative to private care in Australia; the main advantages of going private are reduced waiting periods for elective surgery, the ability to choose your physician and potentially more pleasant care (e.g. use of a private room). Ancillary health services are out-of-hospital services not included in Medicare and are generally private fees^3^. Private ancillaries (or ‘general treatment’) health insurance can be purchased to cover these expenses. The structure of these policies varies, but generally they provide capped coverage for costs associated with dental, replacement corrective eye-wear (diagnostic visits to an optometrist are usually covered by Medicare) and physiotherapy and related treatments. Some more expensive policies also cover naturopathy and remedial massage. As of December 2019, 44% of Australians have some form of private hospital insurance and 53% private ancillaries. The majority of policies are combined hospital/ancillaries policies (83%)^4^.

### Main variables

**Fig. 1:**
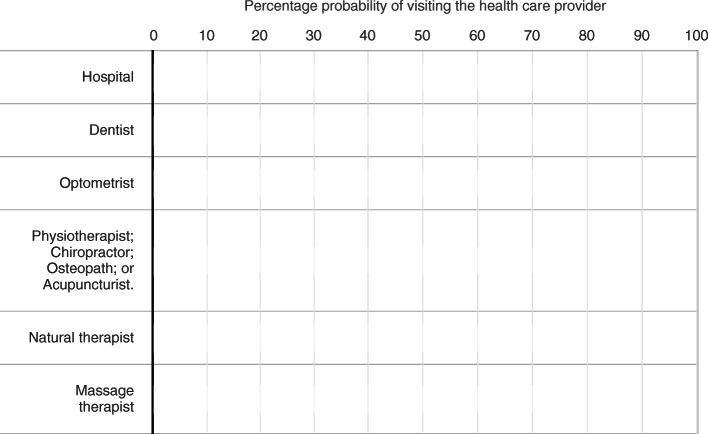
Stated expectations slider question (screenshot)

The key variables for this study are people’s expectations about health service use. Participants in the Online Survey were asked: “*For each health service below, use the slider to indicate how likely (from 0% to 100%) you are to visit this type of health care provider in the next 12 months*” (see Fig. 1). Slider tasks have been shown to reduce the tendency for responses to bunch at 50% compared to open responses [25]. The health services were chosen to match the services that are typically covered by private hospital insurance (hospital admissions) and private ancillaries insurance (dental, optical, physiotherapy (and related), naturopathy and massage). Respondents were only asked about the extensive margin of health service use in part because this matches the information that is collected in HILDA and can therefore be validated^5^.

**Fig. 2:**
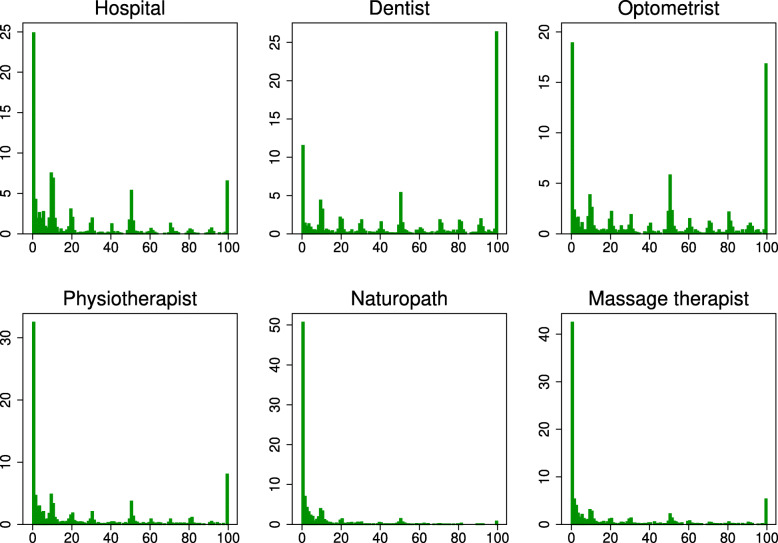
Distribution plots for subjective expectations. Note: Distribution plots show the percentage frequencies in the Online Survey for each subjective expectation of visiting the relevant health service provider in the next 12 months. Participants could respond in units of one percentage point. *n*=1,528

The distributions for the health service expectations (Fig. 2) reflect some common features of these measures, namely bunching at 0%, 50% and 100%, and a tendency for people to round to 5s and 10s [16]^6^. The bunching at 0% and 100% is particularly evident. It is not surprising that many people are certain about visiting a hospital (6.02%), dentist (24.48%), optometrist (15.58%) and physiotherapist^7^ (7.33%) since hospital admissions for elective procedures are known in advance, while it is common for people to have regular scheduled visits to ancillary service providers.

The other key variables are self-reports for whether the person used the relevant health service provider in the last 12 months. These match up with the stated expectation questions with minor exceptions (see Appendix Table A2). The main exceptions are: i) in the Online Survey people are asked about visits to a physiotherapist, chiropractor, osteopath or acupuncturist, while in HILDA they are asked about the first three only; ii) in HILDA, we observe visits to a ‘naturopath, herbalist or acupuncturist’ while in the Online Survey we only observe visits to a naturopath and; iii) we do not observe massage therapist for the HILDA sample. Acupuncturist comprises a trivial fraction of the physiotherapist variable in the Online Survey, so (i) is of minimal concern^8^. Because of (ii) and (iii) I do not consider naturopath and massage therapist when comparing subjective expectations to objective predictions based on HILDA.

Other key variables are the observable characteristics that are common to the Online Survey and HILDA, which are used to build the prediction models. These include characteristics that are likely to reflect preferences, risk and financial means e.g. age, education, sex, employment status, insurance status, risk preferences, household composition, self-assessed health and regional indicators (see Appendix Table A1 for a complete list of variables and definitions). Previous work has established that many of these controls predict hospitalizations [27, 28] and ancillary health service utilization [29], particularly age, gender, income, health and insurance.

One aspect of language in the Online Survey is worth commenting on. When asking people about prior health service use they are asked “*Did you visit any of these health care providers in the last 12 months?*” [Categories: hospital, dentist, optometrist, physiotherapist, chiropractor, osteopath, acupuncturist, naturopath, massage therapist]^9^. This language matches how the question about subjective expectations was asked; however, it is possible for both questions that some people included visits on behalf of another person (for example their child or spouse). If so, it might be more accurate to think of health service use in this study as *contact* with particular service providers (which may or may not involve personal care). In Appendix B I explore this further and show that for most categories, reported health service use is indeed slightly higher in the Online Survey than in HILDA even after adjusting for covariates. In all cases except optometrist this gap disappears when restricting attention to singles without dependent children, suggesting that for this group prior service use reflects personal care only^10^. For this reason I conduct analysis on subjective exceptions using i) the whole sample and ii) separately on singles without dependent children.

## Assessing subjective expectations

Ideally, we would compare people’s subjective expectations to their actual health service use in the future. Since the Online Survey is cross-sectional, this is not possible. Instead I use three common approaches to assess people’s beliefs. First, I compare the aggregate predicted health service use in the next 12 months to the aggregate actual health service use during the last 12 months. Second, I compare the coefficients from models that estimate the past health service use and subjective expectations. Third, I use the HILDA sample to build a prediction model for expected probability of health service use and use this prediction as an objective measure of risk. The correlations between objective risk and subjective expectations are then compared.

### Mean expectations

In Table 2 I compare the mean probability of having used each health service in the last 12 months to the mean subjective probability of using the service in the next 12 months. If there is perfect serial correlation in health service use at the population level, then a necessary condition for well-formed expectations is that mean expected use will equal mean past use. Note however that this condition in not sufficient, since people may form systematically incorrect beliefs which, on average, happen to equal the past incidence^11^.

**Table 2:** Mean realized probabilities vs. expected

	Past	Expected	Diff	*P*-value
	A. All sample (*n*=1,528)			
Hospital	0.317	0.243	0.074	0.000
Dentist	0.573	0.527	0.046	0.004
Optometrist	0.477	0.425	0.052	0.001
Physiotherapist	0.270	0.241	0.029	0.042
Naturopath	0.048	0.089	-0.041	0.000
Massage therapist	0.176	0.186	-0.010	0.411
	B. Childless singles only (*n*=482)			
Hospital	0.255	0.190	0.065	0.005
Dentist	0.527	0.496	0.031	0.288
Optometrist	0.384	0.384	-0.000	0.996
Physiotherapist	0.226	0.218	0.008	0.739
Naturopath	0.039	0.075	-0.035	0.002
Massage therapist	0.139	0.162	-0.023	0.259

Note: ‘Past’ is an indicator for if the person reported visiting the relevant health service provider in the last 12 months. ‘Expected’ is the expected probability of health service use in the next 12 months. *P*-values are based on standard paired t-tests.

Looking at Panel A (full sample), overall the expectations are similar to the past probabilities, although the differences are statistically significant in all but one case (massage therapist). For the main health services (hospital, dentist, optometrist, physiotherapist) average expected use is underestimated compared to past use. The degree ranges from 9% (dentist) to 30% (hospital). The higher discrepancy for naturopath (-46%) could reflect its low frequency. As discussed in the previous section, it is worthwhile separately looking at childless singles, whose responses are less likely to be confounded with health service use by others. For this group, expectations are closer to realizations – only differences for hospital and naturopath are significant.

One unique challenge in interpreting expectations around health services is that these may be influenced by moral hazard. For the insured, expectations are likely to be underpinned by both personal risk as well as anticipated induced usage due to lower price of access. Differences in expectations bias between the insured/uninsured could matter for market outcomes. It is therefore worthwhile considering these groups separately, which I do in Table 3,^12^.

**Table 3:** Mean realized probabilities vs. expected

	Past	Expected	Diff	*P*-value
	A. All privately insured (*n*=751 [766])			
Hospital	0.314	0.239	0.076	0.000
Dentist	0.727	0.676	0.051	0.014
Optometrist	0.594	0.524	0.070	0.002
Physiotherapist	0.359	0.332	0.027	0.215
Naturopath	0.060	0.114	-0.054	0.000
Massage therapist	0.234	0.242	-0.008	0.677
	B. All not privately insured (*n*=777 [762])			
Hospital	0.320	0.248	0.073	0.000
Dentist	0.419	0.377	0.042	0.060
Optometrist	0.360	0.326	0.033	0.122
Physiotherapist	0.180	0.149	0.030	0.070
Naturopath	0.035	0.063	-0.028	0.001
Massage therapist	0.118	0.130	-0.012	0.407
	C. Privately insured childless singles (*n*=194 [201])			
Hospital	0.294	0.190	0.104	0.006
Dentist	0.701	0.682	0.019	0.643
Optometrist	0.527	0.496	0.031	0.481
Physiotherapist	0.348	0.331	0.018	0.679
Naturopath	0.055	0.099	-0.044	0.037
Massage therapist	0.199	0.223	-0.024	0.506
	D. Not privately insured childless singles (*n*=288 [281])			
Hospital	0.229	0.190	0.039	0.190
Dentist	0.402	0.363	0.039	0.282
Optometrist	0.281	0.304	-0.023	0.505
Physiotherapist	0.139	0.138	0.001	0.965
Naturopath	0.028	0.057	-0.029	0.024
Massage therapist	0.096	0.118	-0.022	0.328

Note: See Table 2. Private health insurance means hospital insurance for *n* not in square brackets, and any form of ancillaries insurance for *n* in square brackets.

While the insured tend to utilize ancillary health services more often than the uninsured, there are no strong differences in the gaps between past and expected use by insurance status when focussing on the full sample (Panels A and B). Both groups underestimate their use of the main health services by a similar degree. This is also the case when looking at childless singles (Panels C and D) with one important exception. The underestimation of hospital usage is -35% for the insured compared to -17% (statistically insignificant) for the uninsured. This indicates that the gap may be partly explained by unanticipated *ex post* moral hazard. The fact that we only see this difference for hospitilizations can potentially be explained by the fact that hospitalizations are often due to unexpected health shocks, whereas visits to dentists, optometrists etc. are more likely to be expected^13^.

### Comparing coefficients: subjective expectations vs. realized usage

In this section I compare partial correlations between covariates and past risk and covariates and expectations. If we assume constancy in the partial correlations between covariates and health service from one year to the next, then a necessary condition for accurate expectations is that these correlations are equal^14^. Similarity between the partial correlations would also be consistent with people updating (potentially biased) beliefs like Bayesians in response to knowledge about their risk factors (e.g. health). It could also indicate that beliefs are driven by past health service use, even if past use is a poor predictor of future use. As in the previous section, while concordance is indicative of well-formed beliefs, it is not a sufficient condition.

**Fig. 3:**
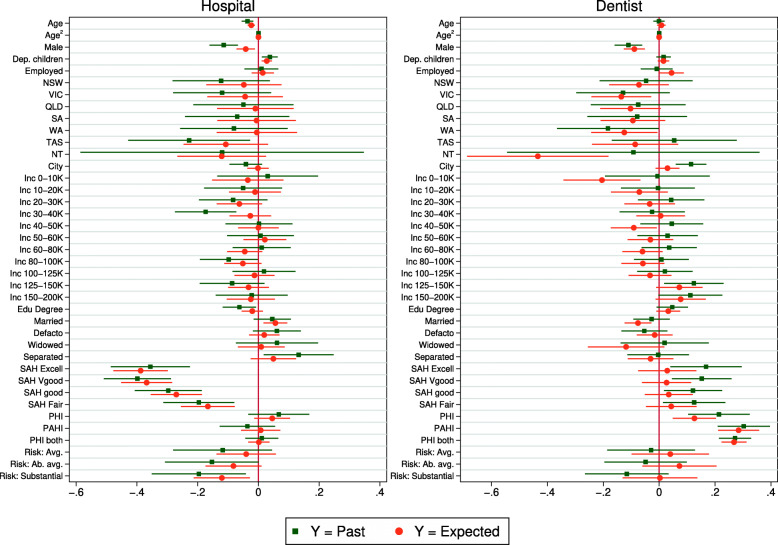
Coefficient estimates – Hospital and dentist use. Note: Displayed are coefficient estimates and 95% confidence intervals (robust standard errors) from linear regression on an indicator for actual health service use in the last 12 months (squares) and expected probability of health service use in the next 12 months (circles). *n*=1,528

**Fig. 4:**
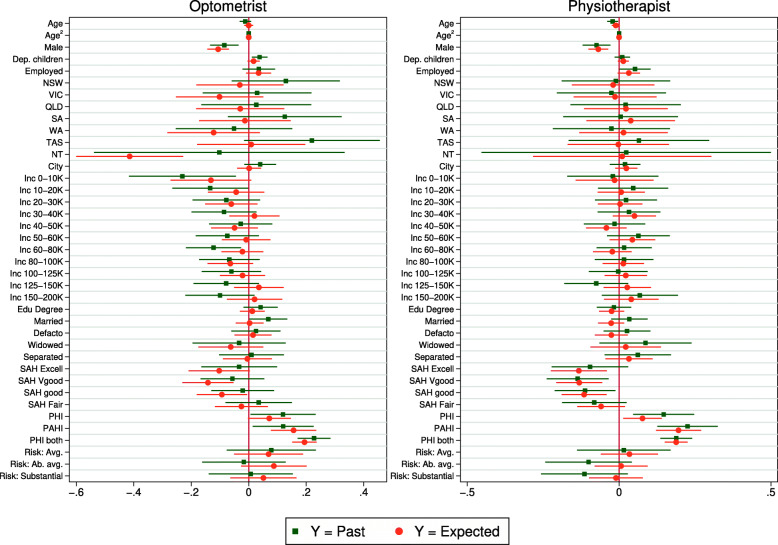
Coefficient estimates – Optometrist and physiotherapist use. Note: See Fig. 3

**Fig. 5:**
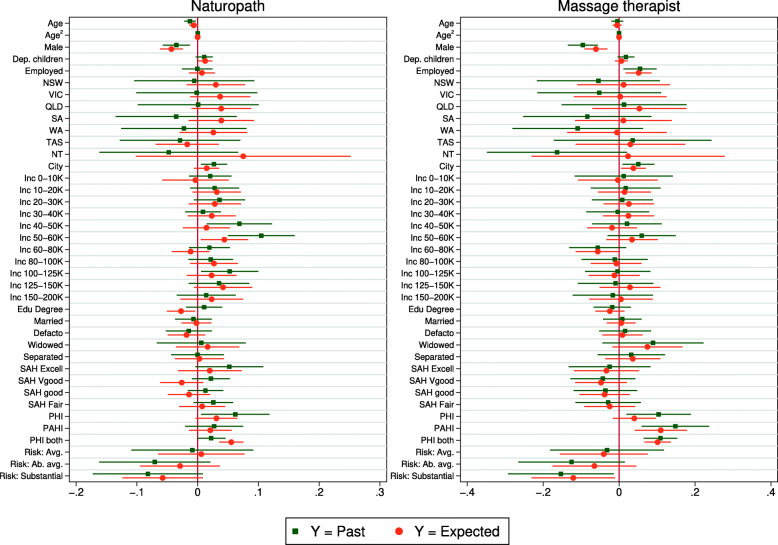
Coefficient estimates – Naturopath and massage therapist use. Note: See Fig. 3

Figures 3, 4 and 5 compare coefficients from linear regression models using the full sample. For each health service, the comparison is between the estimates from a linear probability model on the past realization (e.g. hospital admission) estimated by OLS, and a linear regression on the subjective expectation. The coefficients are also reported in the Online Appendix Tables A3 and A4. For the sake of space, figures for childless singles are relegated to the Appendix (Figs. A1–A3).

Figure 3 considers hospital and dentist. Although tests on the joint equality of the entire vector of coefficients reject equality^15^, the coefficients almost always have the same sign, and when the signs differ usually one or both of the coefficients are statistically insignificant. Major correlates are consistent across models. For example, both past realizations and expectations are concave with respect to age and are significantly negatively signed with respect to being male, health and willingness to take risks. For dentist, correlations with expectations pick up the large partial effects from sex and insurance. Interestingly, the coefficient for male is notably smaller for hospital, which may suggest possible gender effects in the formation of expectations. However, across other health services there is no significant divergence on the male dummy.

Figure 4 looks at optometrist and physiotherapist. Again we see a general pattern of congruence between the estimates and both models suggest the same major predictors (i.e. age, sex, health, insurance). It is a similar story when we look at naturopath in Fig. 5. In this case, age, sex and risk preferences are particularly influential in both models. Finally, the coefficients for massage therapist are strongly correlated; this is the only health service where we cannot reject joint equality of the coefficients (p = 0.151). The superior predictive validity for massage therapist is consistent with results in the previous section.

Results for childless singles are reported in Appendix Figs. A1-A3 and Tables A5 and A6. These results are similar to those for the full sample, although some estimates are less precise, which is expected given the smaller sample. For this group, the joint equality of the coefficients cannot be rejected (at the 5% level) for dentist, optometrist or physiotherapist.

### Correlations between expectations and objective risk

The final exercise to assess expectations compares out-of-sample predictions to subjective expectations. Out-of-sample predictions are generated by estimating a logistic regression model using the HILDA sample to predict health service use in the Online Survey. The prediction model includes all the overlapping covariates in Table A1 and a full factorial of age dummies. These independent variables are all lagged by one year since expectations are for the next 12 months in the Online Survey. To improve predictive power, a penalized likelihood function is maximized using lasso logit regression [30], with the tuning parameter selected using K-fold cross validation and the preferred subset of covariates chosen based on a lowest deviance criterion.

One shortcoming of comparing out-of-sample predictions to expectations is that the correlation is likely to be low if the predictions are poor. On the other hand, difficulty obtaining accurate predictions from observable risk factors adds further weight to the importance of collecting information on subjective expectations. Indeed, despite a large set of covariates and rigorous estimation strategy, the models provide only low-moderate internal predictive power. The pseudo *R*^2^ values range from 0.04 (hospital) to 0.07 (dentist) and the areas under the receiver operating characteristic (ROC) curves are between 0.62-0.68 (see Appendix Fig. A4), slightly below the commonly accepted threshold of 0.7 for moderate predictive power^16^. In exploratory work I added an extensive set of additional health variables available in HILDA covering BMI, diet, exercise, smoking, drinking, social capital, various health conditions, ongoing treatments, mental health and sleep (72 variables in total). Even with this extensive set of controls, the range of pseudo *R*^2^ and ROC values is 0.08-0.09 and 0.69-0.70 respectively, reflecting the difficulty in predicting health service use from survey data, even with detailed health information.

**Fig. 6:**
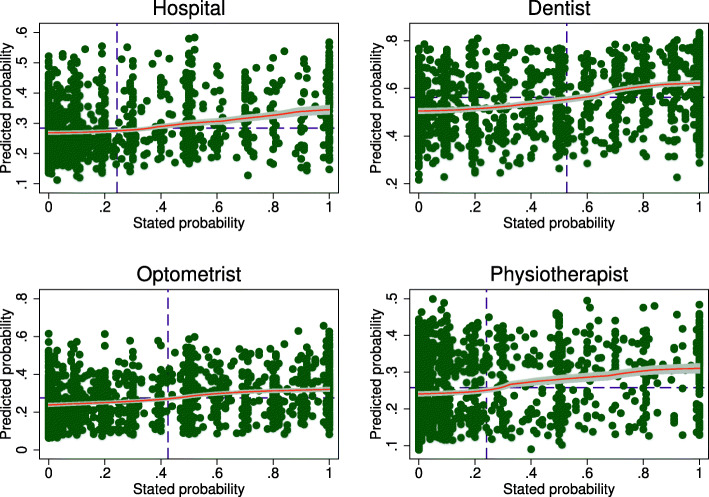
Correlations between subjective and predicted risk. Note: Predicted probabilities are obtained by estimating lasso logit models on an indicator for actual health service use in the last 12 months using the HILDA sample and predicting health service use in the Online Sample from the resulting estimates. Stated expectations are the expected probability of health service use in the next 12 months. Dashed lines indicate the mean values for each axis. Data from the 2013 wave of HILDA are used for the prediction models. After restricting the sample to those aged 25-64 years with non-missing data, *n*=9,460, *n*=9,306, *n*=9,362 and *n*=9,460 for hospital, dentist, optometrist and physiotherapist respectively. *n*=1,528 in the Online Survey sample

**Fig. 7:**
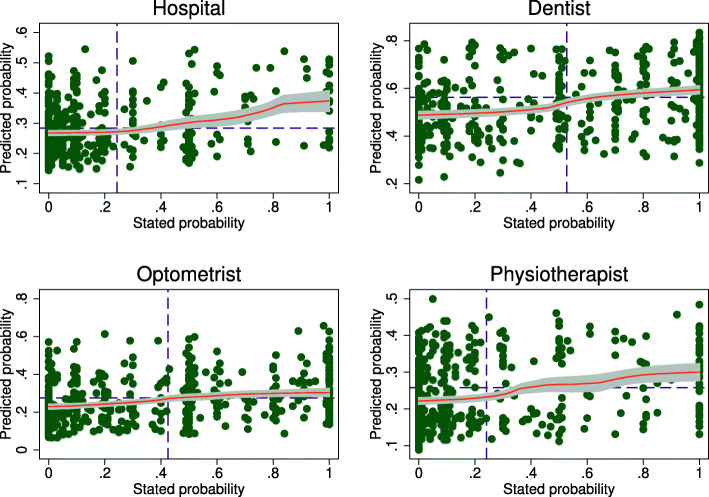
Correlations between subjective and predicted risk for childless singles. Note: See Fig. 6. *n*=482 childless singles in the Online Survey

Figure 6 reports scatter plots and local polynomial fits between the HILDA predictions and stated expectations for the full sample and Fig. 7 reports the same correlations for childless singles only. In all cases the correlations are positive, with the following Pearson correlation coefficients for the full sample (childless singles): hospital = 0.30 (0.32); dentist = 0.39 (0.39); optometrist = 0.33 (0.29); and physiotherapist = 0.28 (0.32). While these correlations are not overly strong, they need to be evaluated against the low predictive power of the lasso logit models. Subjective expectations do seem to meaningfully correlate with an objective measure of risk^17^.

**Fig. 8:**
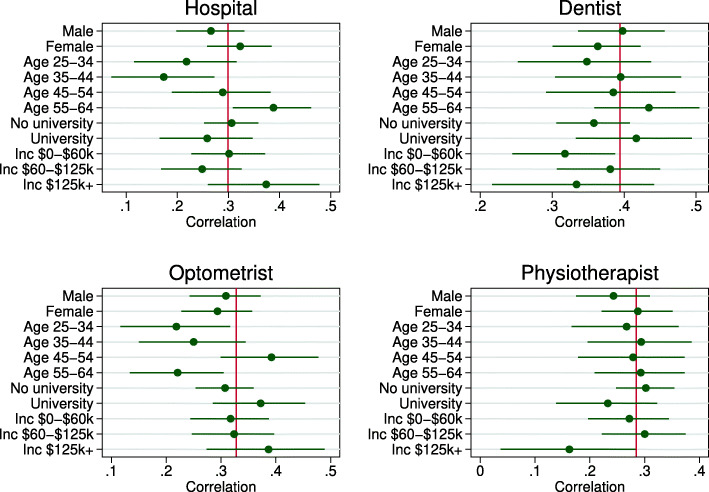
Correlations between subjective and predicted risk by subgroup. Note: Each panel shows the Pearson correlation and 95% confidence interval for subjective expectations and predicted risk (see Fig. 6) for each health service by demographic subgroup. Vertical lines are the correlations for the whole sample

Figure 8 shows how the Pearson correlations vary by sex, age, education and household income. This analysis may reveal groups with more or less accurate beliefs; however, it may also capture variation in the quality of objective predictions (moreover, correlation does not directly assess accuracy). While in some individual cases groups stand-out (for example, people aged 55-64 years have a stronger association for hospitalization and a weaker association for optometrist), there is no systematic pattern of any particular group correlating more or less strongly across the spectrum of health services.

### Summary

Subjective expectations closely reflect patterns of observed utilization, are predicted by the same covariates as observed utilization, and correlate with objective measures of risk. There is a moderate tendency towards underestimating risk on average for the highest use health services (hospital, dentist, optometrist, physiotherapist). This bias may be partly due to phrasing in the Online Survey leading to some reported health service use being on behalf of family members; for childless singles, the differences largely disappear. The mean expected hospitalization probability is also closer to past hospitalization for childless singles without private health insurance, which could indicate the privately insured experience unanticipated moral hazard. Overall, the results support subjective expectations as a high-quality single control for health service risk^18^. The poor performance of observable risk factors in predicting utilization further supports the collection of subjective expectations data.

## Using subjective expectations to predict insurance take-up

One of the main purported benefits of subjective expectations data is that they can add informational value to econometric decision models [16]. This is especially true when expectations differ from objective probabilities, or when objective probabilities are difficult to estimate.

To further explore the informational value in subjective expectations over health service usage, I test whether they can predict private health insurance take-up, even after conditioning on objective risk factors (including past health service use). This is a natural outcome variable in this setting since the health services were specifically chosen to predict insurance choices. Theoretically, expectations should positively correlate with insurance coverage for two reasons. First, those at higher risk have more to gain from insurance (adverse selection). Second, insurance reduces the cost of (and therefore increases demand for) health care (moral hazard). However, this prediction is more complicated in the case of private hospital insurance in Australia since Medicare offers a free alternative to private care. In this setting, theoretical models posit that the ‘quality gap’ in care drives demand for insurance [31]. Since preferences for higher quality care (e.g. shorter waiting times for procedures) may be inversely related to risk of hospitalization, it is less clear whether risk will be positively correlated with coverage. Indeed, previous research has actually found that people favorably select into private hospital insurance [27, 32].

To begin I focus on the probability of purchasing private hospital insurance and estimate three linear probability models: (1) controlling only for subjective expectations; (2) expectations and prior health service use; (3) expectations, prior health service use and the controls listed in Table A1 (see Table 4). Consistent with previous work, there is evidence of advantageous selection in that those more likely to visit a hospital are actually less likely to have private insurance. This estimate remains significant even after conditioning on previous health service use and other controls.

**Table 4:** LPM estimates: Has private hospital insurance

	(1)	(2)	(3)
Hospital expectation	-0.155^***^	-0.125^***^	-0.088^**^
	(0.039)	(0.046)	(0.044)
Dentist expectation	0.322^***^	0.236^***^	0.176^***^
	(0.035)	(0.041)	(0.040)
Optometrist expectation	0.111^***^	0.061	0.075^**^
	(0.037)	(0.039)	(0.037)
Physiotherapist expectation	0.174^***^	0.119^**^	0.125^**^
	(0.045)	(0.058)	(0.054)
Naturopath expectation	0.191^**^	0.205^**^	0.205^**^
	(0.078)	(0.089)	(0.083)
Massage expectation	0.011	-0.031	-0.070
	(0.051)	(0.070)	(0.068)
Dentist visit		0.114^***^	0.074^**^
		(0.031)	(0.029)
Optometrist visit		0.119^***^	0.090^***^
		(0.027)	(0.026)
Physiotherapist visit		0.042	0.031
		(0.039)	(0.036)
Naturopath visit		-0.044	-0.083
		(0.069)	(0.066)
Massage visit		0.043	0.052
		(0.050)	(0.048)
Hospital visit		-0.017	0.013
		(0.030)	(0.028)
Other controls?	No	No	Yes
Observations	1,528	1,528	1,528

Note: The dependent variable is an indicator for being covered by a private hospital insurance policy (mean = 0.497). The expectations variables are the stated probability of health service use in the next 12 months. The health service visit variables are indicators for having visited the relevant health care provider in the previous 12 months. Other controls are detailed in Table A1. Robust standard errors in parentheses. ^*^ *p*<0.10, ^**^ *p*<0.05, ^***^ *p*<0.01

Next I turn to predicting private ancillaries health insurance (Table 5). Since most people purchase bundled hospital/ancillaries cover, there is considerable overlap in these dependent variables (77% of people in the Online Survey with any type of private cover have bundled cover). This time, expectations are positively correlated with coverage, as predicted by standard theory. Ancillaries are not covered by Medicare, so this result is unsurprising. Importantly, even after conditioning on prior use and other observable risk factors, expectations for the major services covered by this type of insurance (dentist, optometrist, physiotherapist) independently predict demand for insurance and are highly significant.

**Table 5:** LPM estimates: Has private ancillaries insurance

	(1)	(2)	(3)
Hospital expectation	-0.191^***^	-0.158^***^	-0.120^***^
	(0.039)	(0.045)	(0.044)
Dentist expectation	0.383^***^	0.297^***^	0.235^***^
	(0.034)	(0.041)	(0.039)
Optometrist expectation	0.122^***^	0.078^**^	0.087^**^
	(0.035)	(0.038)	(0.037)
Physiotherapist expectation	0.252^***^	0.209^***^	0.196^***^
	(0.045)	(0.058)	(0.054)
Naturopath expectation	0.081	0.124	0.122
	(0.072)	(0.082)	(0.078)
Massage expectation	0.042	-0.001	-0.030
	(0.048)	(0.067)	(0.062)
Dentist visit		0.112^***^	0.079^***^
		(0.030)	(0.028)
Optometrist visit		0.103^***^	0.077^***^
		(0.027)	(0.026)
Physiotherapist visit		0.032	0.030
		(0.038)	(0.036)
Naturopath visit		-0.086	-0.110^*^
		(0.064)	(0.061)
Massage visit		0.042	0.047
		(0.048)	(0.044)
Hospital visit		-0.026	-0.011
		(0.030)	(0.028)
Other controls?	No	No	Yes
Observations	1,528	1,528	1,528

Note: The dependent variable is an indicator for being covered by a private ancillaries insurance policy (mean = 0.548). See Table 4 for further details.

## Conclusion

This paper provides evidence that people’s subjective expectations over broadly defined, common health services, such as hospitalizations and visits to dentists and optometrists, are an informative measure of their actual risk. Questions on expectations could be included in standard household surveys at minimal cost.

One important policy implication of this work is that consumers’ hold valuable private information over their health service risk. While panel data would be needed to firmly assess whether beliefs are accurate, my analysis is indicative that choice inconsistencies in health service related markets, such as consumers failing to select into optimal health insurance plans [33], may be due to reasons other than biased beliefs.

There are some limitations of this study worth noting. It is a single study, in a particular institutional environment, and considers a particular set of health services. Some use of the services will be for preventative and scheduled care; it is likely that expectations over health services with less predictability would be less accurate. Testing the generalizability of the results to other groups of services, in other institutional settings, would therefore be worthwhile.

## Supplementary Information

**Additional file 1** Online Appendix: The informational content of subjective expectations for health service use

## Data Availability

Data from the Online Survey and replication code is available with the Supplementary Material. Researchers wishing to use the HILDA data can apply for these data through the Australian Data Archive (https://dataverse.ada.edu.au/). Declarations

## Footnotes

1 Although not strictly health service use, [4] provide evidence that subjective expectations for long-term care use convey meaningful information about risk that is independent of objective risk scores. [18] asked people about their expected probability of getting a swine flu vaccination. They did not assess the accuracy of this expectation but did show that it positively correlated with perceived risk of contracting swine flu.

2 A copy of the survey is provided as part of the Supplementary Material.

3 It is possible to get some reimbursements through allied health care plans, which allow partial Medicare assistance for a limited number of visits to ancillary health service providers at the direction of a general practitioner.

4 Industry statistics are available at https://www.apra.gov.au/industries/private-health-insurance

5 These binary measures are also available in other major Australian health surveys e.g. the National Health Survey, the Australian Longitudinal Survey of Women’s Health and 45 and Up. There may be additional value in eliciting expectations around the intensive margin, as well as expected expenses and their distribution; this is left for future work.

6 I do not address measurement error due to rounding in this study. In principle, it may be possible to learn something about bunching by exploiting the fact that there are multiple stated probability questions. The intuition is that we can learn about a person’s tendency to round by observing their repeated choices (see e.g. [26]).

7 For brevity, I will simply use physiotherapist to refer to the collective of physiotherapist, chiropractor, osteopath and acupuncturist hereafter.

8 Only five people in the Online Survey visited an acupuncturist in the last 12 months and did not also visit a physiotherapist, chiropractor or osteopath.

9 They were also asked about how many visits they had. However, subjective expectations were only elicited for the probability of any visit, so I do not use this information.

10 The persistent gap for optometrist may be due to more restrictive wording in HILDA, as discussed in Appendix B.

11 One way this could occur is if people base expected future use on past use, even if past use has little bearing on future use. In the extreme case, past use would perfectly correlate with expectations (it is clear from Fig. 2 this is not the case, as most people are uncertain about future use).

12 Expectations may also differ because the insured anticipate shorter waiting periods for hospital care. However, longer waiting periods for public care will also mean that the uninsured should have higher residual expectations for hospital care not yet received from health shocks in previous periods. On net, these effects should cancel out. It may be worthwhile in future work to include an additional subjective expectations question about only expected hospitalizations due to future events, and to jointly elicit expected waiting periods.

13 Nevertheless, the fact that the majority of people report expected health service usage between 1%-99% (see Fig. 2) indicates that there is considerable uncertainty, even for these services.

14 We would also require the means to be the same, which would imply equal intercept terms.

15 This was tested using Stata’s *suest* command for a generalized Hausman test, with the intercepts excluded from the coefficient vector.

16 The ROC curves show how the fraction of correctly identified positive outcome cases (sensitivity) evolves as one minus the fraction of correctly identified negative outcome cases (specificity) increases. The area between the ROC curve and the 45 degree line (the ROC curve in a model with no predictive power) gives a measure of model fit ranging from 0 to 1, with higher values indicating better fit.

17 Figure 6 also shows fairly strong concordance between mean predicted and stated probabilities. An exception is for optometry, which may be due to more restrictive wording in HILDA than in the Online Survey (see Appendix B).

18 While noting that the efficacy of subjective expectations is not necessarily tied to their ability to predict actual risk (consumers are assumed to act on expectations regardless of whether they are biased).
