# Supplementary material for: The informational content of subjective expectations for health service use
Source: BMC Health Serv Res. 2021 May 17;21:464. doi: 10.1186/s12913-021-06464-7 (PMC8130259; doi:10.1186/s12913-021-06464-7)
Supplement: Supplementary file 1 — Additional file 1 Online Appendix: The informational content of subjective expectations for health service use [file 12913_2021_6464_MOESM1_ESM.pdf]

Online Appendix: The informational content of subjective expectations for health service use

A Additional Tables and Figures

Table A1: Control variables in the Online Survey common to HILDA

Variable	Definition
Age	Age in years
Male	=1 if male
Dep. children	Number of dependent children. A dependent child is classified as a child aged under 18 years (or under 24 years if studying fulltime) who relies on you for maintenance
Employed	=1 if employed (worked in a job, business or farm (or was on some sort of leave from a a job, business or farm) in the last 7 days)
NSW	=1 if lives in New South Wales
VIC	=1 if lives in Victoria
QLD	=1 if lives in Queensland
SA	=1 if lives in South Australia
WA	=1 if lives in Western Australia
TAS	=1 if lives in Tasmania
NT	=1 if lives in Northern Territory
City	=1 if lives in a major city (Sydney, Melbourne, Brisbane, Adelaide, Perth, Canberra)
Inc 0-10K	=1 if household income from all sources for the previous 12 months before tax and other deductions is between \$0–\$9,999
Inc 10-20K	=1 if household income is between \$10,000–\$19,999
Inc 20-30K	=1 if household income is between \$20,000–\$29,999
Inc 30-40K	=1 if household income is between \$30,000–\$39,999
Inc 40-50K	=1 if household income is between \$40,000–\$49,999
Inc 50-60K	=1 if household income is between \$50,000–\$59,999
Inc 60-80K	=1 if household income is between \$60,000–\$79,999
Inc 80-100K	=1 if household income is between \$80,000–\$99,999
Inc 100-125K	=1 if household income is between \$100,000–\$124,999
Inc 125-150K	=1 if household income is between \$125,000–\$149,999
Inc 150-200K	=1 if household income is between \$150,000–\$199,999
Inc 200K+	=1 if household income is between \$200,000 or more
Inc missing	=1 if household income is missing
Edu Degree	=1 if highest level of education is bachelors degree or higher
Married	=1 if married
Defacto	=1 if in a de-facto relationship
Widowed	=1 if widowed

Separated	=1 if separated
SAH Excell	=1 if self-assessed health 'excellent'
SAH Vgood	=1 if self-assessed health 'very good'
SAH good	=1 if self-assessed health 'good'
SAH Fair	=1 if self-assessed health 'fair'
SAH poor	=1 if self-assessed health 'poor'
PHI	=1 if has private hospital insurance only
PAHI	=1 if has private ancillaries (general treatment) insurance only
PHI both	=1 if has combined private hospital/ancillaries insurance
Risk: None	=1 if self-assessed willingness to take financial risks 'not willing to take any risks'
Risk: Avg.	=1 if self-assessed willingness to take financial risks 'average'
Risk: Ab. avg.	=1 if self-assessed willingness to take financial risks 'above average'
Risk: Substantial	=1 if self-assessed willingness to take financial risks 'substantial'

Table A2: Health service measures in HILDA

Hospital	Admitted to hospital as a day or night patient, or saw a hospital doctor for outpatient or emergency care in last 12 months
Dentist	How long has it been since you last saw a dentist? [0-12 months]
Optometrist	During the last 12 months, have you seen any of these types of health care providers about your health? [Optometrist]
Physiotherapist	During the last 12 months, have you seen any of these types of health care providers about your health? [Physiotherapist, chiropractor or osteopath]
Naturopath	During the last 12 months, have you seen any of these types of health care providers about your health? [Alternative health practitioner, such as a naturopath, acupuncturist or herbalist]
Massage therapist	N/A

Table A3: Linear regressions on use and expectations

	Hospital		Dentist		Optometrist	
	Past	Expected	Past	Expected	Past	Expected
Age	-0.036***	-0.023***	-0.001	0.008	-0.012	-0.000
	(0.010)	(0.006)	(0.010)	(0.008)	(0.010)	(0.008)
Age^2	0.000***	0.000***	0.000	-0.000	0.000**	0.000
	(0.000)	(0.000)	(0.000)	(0.000)	(0.000)	(0.000)
Male	-0.114***	-0.041***	-0.110***	-0.089***	-0.085***	-0.106***
	(0.024)	(0.015)	(0.025)	(0.019)	(0.025)	(0.019)
Dep. children	0.038***	0.028***	0.016	0.016	0.038***	0.017

	(0.013)	(0.009)	(0.013)	(0.010)	(0.014)	(0.011)
Employed	0.010	0.014	-0.009	0.045**	0.035	0.034
	(0.028)	(0.018)	(0.029)	(0.022)	(0.029)	(0.022)
NSW	-0.122	-0.048	-0.047	-0.072	0.129	-0.031
	(0.082)	(0.063)	(0.085)	(0.054)	(0.096)	(0.077)
VIC	-0.119	-0.044	-0.129	-0.135**	0.029	-0.101
	(0.082)	(0.063)	(0.085)	(0.054)	(0.096)	(0.077)
QLD	-0.049	-0.010	-0.075	-0.102*	0.026	-0.029
	(0.084)	(0.064)	(0.087)	(0.055)	(0.097)	(0.078)
SA	-0.070	-0.006	-0.078	-0.094	0.126	-0.013
	(0.087)	(0.066)	(0.091)	(0.059)	(0.101)	(0.081)
WA	-0.080	-0.005	-0.183**	-0.125**	-0.051	-0.122
	(0.090)	(0.067)	(0.093)	(0.060)	(0.103)	(0.082)
TAS	-0.228**	-0.107	0.054	-0.086	0.220*	0.009
	(0.102)	(0.071)	(0.114)	(0.079)	(0.120)	(0.096)
NT	-0.119	-0.121	-0.092	-0.434***	-0.102	-0.414***
	(0.238)	(0.074)	(0.230)	(0.129)	(0.222)	(0.095)
City	-0.041	-0.001	0.114***	0.030	0.040	0.001
	(0.027)	(0.018)	(0.028)	(0.022)	(0.028)	(0.021)
Inc 0-10K	0.031	-0.035	-0.006	-0.204***	-0.231**	-0.131*
	(0.085)	(0.060)	(0.096)	(0.070)	(0.095)	(0.072)
Inc 10-20K	-0.050	-0.011	-0.004	-0.071	-0.134**	-0.044
	(0.065)	(0.043)	(0.067)	(0.052)	(0.067)	(0.050)
Inc 20-30K	-0.083	-0.062	0.043	-0.034	-0.078	-0.061
	(0.058)	(0.038)	(0.060)	(0.046)	(0.060)	(0.046)
Inc 30-40K	-0.174***	-0.026	-0.025	0.005	-0.086	0.020
	(0.052)	(0.035)	(0.059)	(0.044)	(0.058)	(0.044)
Inc 40-50K	0.002	0.000	0.045	-0.091**	-0.028	-0.049
	(0.056)	(0.034)	(0.057)	(0.042)	(0.056)	(0.041)
Inc 50-60K	0.007	0.021	0.030	-0.031	-0.074	-0.009
	(0.056)	(0.036)	(0.055)	(0.042)	(0.056)	(0.043)
Inc 60-80K	0.011	-0.045	0.036	-0.060	-0.123**	-0.022
	(0.049)	(0.030)	(0.050)	(0.037)	(0.049)	(0.037)
Inc 80-100K	-0.097**	-0.051	0.008	-0.058	-0.067	-0.064
	(0.048)	(0.031)	(0.050)	(0.039)	(0.054)	(0.040)
Inc 100-125K	0.019	-0.013	0.021	-0.033	-0.060	-0.021
	(0.053)	(0.034)	(0.050)	(0.039)	(0.053)	(0.040)
Inc 125-150K	-0.086	-0.032	0.124**	0.072*	-0.078	0.035
	(0.054)	(0.034)	(0.054)	(0.042)	(0.058)	(0.044)
Inc 150-200K	-0.022	-0.025	0.112*	0.077*	-0.100	0.020
	(0.060)	(0.040)	(0.058)	(0.046)	(0.061)	(0.049)
Edu Degree	-0.062**	-0.020	0.047	0.033	0.041	0.013
	(0.028)	(0.018)	(0.029)	(0.022)	(0.030)	(0.022)

Married	0.046	0.056***	-0.027	-0.076***	0.068**	0.003
	(0.031)	(0.020)	(0.033)	(0.024)	(0.034)	(0.025)
Defacto	0.061	0.019	-0.053	-0.016	0.025	0.015
	(0.040)	(0.025)	(0.042)	(0.033)	(0.044)	(0.033)
Widowed	0.061	0.009	0.020	-0.118*	-0.033	-0.062
	(0.069)	(0.039)	(0.080)	(0.070)	(0.083)	(0.058)
Separated	0.133**	0.049	-0.003	-0.031	0.010	-0.005
	(0.059)	(0.038)	(0.056)	(0.042)	(0.058)	(0.043)
SAH Excell	-0.356***	-0.388***	0.168***	0.029	-0.033	-0.103*
	(0.067)	(0.046)	(0.065)	(0.053)	(0.067)	(0.054)
SAH Vgood	-0.398***	-0.368***	0.153***	0.027	-0.057	-0.142***
	(0.056)	(0.043)	(0.054)	(0.045)	(0.057)	(0.045)
SAH good	-0.297***	-0.270***	0.121**	0.034	-0.021	-0.093**
	(0.056)	(0.043)	(0.053)	(0.044)	(0.056)	(0.045)
SAH Fair	-0.196***	-0.166***	0.125**	0.043	0.035	-0.025
	(0.060)	(0.045)	(0.057)	(0.046)	(0.059)	(0.047)
PHI	0.067	0.046	0.214***	0.126***	0.119**	0.072*
	(0.051)	(0.030)	(0.056)	(0.039)	(0.058)	(0.038)
PAHI	-0.036	0.008	0.303***	0.284***	0.120**	0.156***
	(0.046)	(0.033)	(0.048)	(0.037)	(0.054)	(0.040)
PHI both	0.011	0.002	0.272***	0.268***	0.227***	0.194***
	(0.028)	(0.018)	(0.029)	(0.023)	(0.029)	(0.022)
Risk: Avg.	-0.117	-0.040	-0.029	0.040	0.079	0.069
	(0.083)	(0.050)	(0.080)	(0.070)	(0.079)	(0.061)
Risk: Ab. avg.	-0.153*	-0.082*	-0.048	0.073	-0.017	0.087
	(0.079)	(0.047)	(0.075)	(0.068)	(0.074)	(0.058)
Risk: Substantial	-0.196**	-0.120**	-0.116	0.003	0.007	0.051
	(0.079)	(0.047)	(0.076)	(0.068)	(0.075)	(0.059)
Constant	1.694***	1.084***	0.290	0.177	0.348	0.263
	(0.251)	(0.163)	(0.259)	(0.191)	(0.265)	(0.199)
Observations	1,528	1,528	1,528	1,528	1,528	1,528
R^2	0.106	0.148	0.165	0.217	0.142	0.167
Hausman χ^2 (P-val)	(0.004)		(0.002)		(0.000)	

Note: For each health service, the left column reports OLS estimates on a dummy for whether the person visited the relevant health care provider in the last 12 months. The right column reports OLS estimates on the subjective probability of visiting the relevant health care provider in the next 12 months. The Hausman tests are on the joint equality of the left and right column coefficients excluding constants. Robust standard errors in parentheses. * $p < 0.10$, ** $p < 0.05$, *** $p < 0.01$

Table A4: Linear regressions on use and expectations

	Physiotherapist		Naturopath		Massage	
	Past	Expected	Past	Expected	Past	Expected

Age	-0.021**	-0.011	-0.013***	-0.006*	-0.005	-0.005
	(0.009)	(0.007)	(0.005)	(0.004)	(0.008)	(0.006)
Age^2	0.000**	0.000	0.000**	0.000	0.000	0.000
	(0.000)	(0.000)	(0.000)	(0.000)	(0.000)	(0.000)
Male	-0.074***	-0.068***	-0.035***	-0.043***	-0.095***	-0.061***
	(0.023)	(0.017)	(0.011)	(0.010)	(0.020)	(0.016)
Dep. children	0.010	0.014	0.011	0.012**	0.018	0.006
	(0.013)	(0.009)	(0.007)	(0.006)	(0.011)	(0.008)
Employed	0.053**	0.032*	-0.001	0.007	0.055**	0.051***
	(0.026)	(0.019)	(0.013)	(0.011)	(0.022)	(0.017)
NSW	-0.011	-0.020	-0.006	0.030	-0.054	0.012
	(0.091)	(0.069)	(0.050)	(0.025)	(0.083)	(0.062)
VIC	-0.025	-0.013	-0.001	0.037	-0.052	0.003
	(0.092)	(0.070)	(0.051)	(0.025)	(0.083)	(0.062)
QLD	0.022	0.023	0.001	0.039	0.013	0.054
	(0.093)	(0.070)	(0.051)	(0.025)	(0.084)	(0.063)
SA	0.005	0.038	-0.035	0.039	-0.083	0.011
	(0.096)	(0.074)	(0.051)	(0.028)	(0.086)	(0.065)
WA	-0.025	0.015	-0.023	0.026	-0.109	-0.005
	(0.098)	(0.074)	(0.053)	(0.028)	(0.088)	(0.067)
TAS	0.065	-0.003	-0.029	-0.017	0.036	0.030
	(0.118)	(0.085)	(0.051)	(0.027)	(0.106)	(0.074)
NT	0.023	0.010	-0.048	0.075	-0.163*	0.024
	(0.243)	(0.150)	(0.058)	(0.090)	(0.094)	(0.130)
City	0.020	0.024	0.027**	0.015	0.051**	0.038**
	(0.026)	(0.019)	(0.011)	(0.011)	(0.021)	(0.016)
Inc 0-10K	-0.021	-0.015	0.021	-0.004	0.012	-0.003
	(0.077)	(0.066)	(0.018)	(0.028)	(0.066)	(0.054)
Inc 10-20K	0.046	0.007	0.028	0.032	0.017	0.014
	(0.059)	(0.040)	(0.021)	(0.020)	(0.047)	(0.036)
Inc 20-30K	0.023	0.003	0.036*	0.028	0.009	0.026
	(0.052)	(0.037)	(0.021)	(0.022)	(0.041)	(0.034)
Inc 30-40K	0.033	0.051	0.009	0.023	-0.004	0.025
	(0.053)	(0.036)	(0.015)	(0.020)	(0.042)	(0.034)
Inc 40-50K	-0.015	-0.042	0.069**	0.015	0.021	-0.018
	(0.052)	(0.034)	(0.027)	(0.020)	(0.047)	(0.033)
Inc 50-60K	0.064	0.044	0.105***	0.044**	0.060	0.034
	(0.053)	(0.038)	(0.028)	(0.020)	(0.046)	(0.035)
Inc 60-80K	0.017	-0.022	0.019	-0.011	-0.056	-0.056*
	(0.046)	(0.032)	(0.017)	(0.016)	(0.038)	(0.030)
Inc 80-100K	0.016	0.014	0.022	0.027	-0.011	-0.007
	(0.049)	(0.035)	(0.019)	(0.020)	(0.044)	(0.034)
Inc 100-125K	-0.003	0.022	0.053**	0.023	-0.004	-0.013

	(0.049)	(0.035)	(0.024)	(0.021)	(0.044)	(0.034)
Inc 125-150K	-0.075	0.027	0.035	0.042*	-0.009	0.029
	(0.054)	(0.040)	(0.025)	(0.025)	(0.051)	(0.041)
Inc 150-200K	0.068	0.040	0.014	0.023	-0.016	0.005
	(0.064)	(0.046)	(0.025)	(0.026)	(0.054)	(0.043)
Edu Degree	-0.017	-0.025	0.011	-0.027**	-0.018	-0.024
	(0.029)	(0.021)	(0.015)	(0.012)	(0.025)	(0.019)
Married	0.034	-0.026	-0.007	-0.002	0.009	0.006
	(0.031)	(0.022)	(0.015)	(0.013)	(0.026)	(0.019)
Defacto	0.026	-0.025	-0.015	-0.018	0.016	0.009
	(0.039)	(0.028)	(0.019)	(0.016)	(0.035)	(0.027)
Widowed	0.087	0.022	0.006	0.016	0.089	0.075
	(0.077)	(0.059)	(0.037)	(0.027)	(0.068)	(0.047)
Separated	0.062	0.033	-0.000	0.003	0.032	0.036
	(0.056)	(0.040)	(0.022)	(0.021)	(0.045)	(0.037)
SAH Excell	-0.096	-0.132***	0.052*	0.020	-0.025	-0.033
	(0.064)	(0.047)	(0.029)	(0.027)	(0.055)	(0.044)
SAH Vgood	-0.136***	-0.131***	0.022	-0.026	-0.043	-0.047
	(0.052)	(0.038)	(0.016)	(0.018)	(0.044)	(0.035)
SAH good	-0.112**	-0.115***	0.013	-0.014	-0.036	-0.038
	(0.051)	(0.038)	(0.015)	(0.018)	(0.043)	(0.034)
SAH Fair	-0.082	-0.059	0.026	0.007	-0.029	-0.025
	(0.054)	(0.040)	(0.017)	(0.019)	(0.044)	(0.034)
PHI	0.147***	0.077**	0.062**	0.031*	0.104**	0.040
	(0.051)	(0.032)	(0.029)	(0.018)	(0.043)	(0.029)
PAHI	0.226***	0.196***	0.027	0.021	0.148***	0.110***
	(0.051)	(0.038)	(0.024)	(0.018)	(0.045)	(0.035)
PHI both	0.188***	0.188***	0.022*	0.055***	0.109***	0.102***
	(0.027)	(0.019)	(0.012)	(0.010)	(0.023)	(0.018)
Risk: Avg.	0.016	0.034	-0.009	0.006	-0.032	-0.040
	(0.078)	(0.048)	(0.051)	(0.036)	(0.076)	(0.059)
Risk: Ab. avg.	-0.101	0.007	-0.071	-0.029	-0.125*	-0.065
	(0.073)	(0.044)	(0.047)	(0.034)	(0.072)	(0.056)
Risk: Substantial	-0.114	-0.010	-0.082*	-0.058*	-0.153**	-0.121**
	(0.073)	(0.045)	(0.046)	(0.034)	(0.071)	(0.056)
Constant	0.748***	0.463***	0.334***	0.226**	0.405*	0.358**
	(0.241)	(0.176)	(0.126)	(0.095)	(0.209)	(0.162)
Observations	1,528	1,528	1,528	1,528	1,528	1,528
R^2	0.082	0.119	0.078	0.086	0.087	0.092
Hausman χ^2 (P-val)	(0.009)		(0.004)		(0.151)	

Note: See Table A3.

Table A5: Linear regressions on use and expectations: Childless singles only

	Hospital		Dentist		Optometrist	
	Past	Expected	Past	Expected	Past	Expected
Age	-0.030*	-0.011	-0.027	0.011	-0.012	0.013
	(0.015)	(0.009)	(0.017)	(0.012)	(0.017)	(0.013)
Age^2	0.000**	0.000	0.000*	-0.000	0.000	-0.000
	(0.000)	(0.000)	(0.000)	(0.000)	(0.000)	(0.000)
Male	-0.096**	-0.076***	-0.103**	-0.120***	-0.087*	-0.116***
	(0.044)	(0.027)	(0.048)	(0.037)	(0.048)	(0.035)
Employed	-0.004	-0.047	-0.032	0.017	0.024	0.032
	(0.050)	(0.031)	(0.054)	(0.043)	(0.052)	(0.043)
NSW	-0.121	-0.008	-0.256**	-0.191***	0.124	-0.027
	(0.150)	(0.092)	(0.107)	(0.068)	(0.168)	(0.152)
VIC	-0.107	0.008	-0.319***	-0.259***	0.060	-0.098
	(0.150)	(0.094)	(0.107)	(0.068)	(0.169)	(0.153)
QLD	0.002	-0.031	-0.192*	-0.223***	0.073	-0.017
	(0.157)	(0.095)	(0.112)	(0.074)	(0.170)	(0.154)
SA	0.043	0.003	-0.250**	-0.195**	0.169	-0.060
	(0.164)	(0.100)	(0.127)	(0.084)	(0.178)	(0.160)
WA	-0.107	-0.012	-0.385***	-0.312***	-0.068	-0.198
	(0.163)	(0.102)	(0.127)	(0.086)	(0.177)	(0.158)
TAS	-0.200	-0.087	-0.055	-0.206	0.323	0.119
	(0.198)	(0.107)	(0.164)	(0.134)	(0.211)	(0.171)
NT	-0.134	-0.024	-0.405	-0.610***	-0.274	-0.320*
	(0.201)	(0.111)	(0.258)	(0.106)	(0.241)	(0.174)
City	-0.032	-0.004	0.055	0.026	0.066	0.006
	(0.045)	(0.027)	(0.050)	(0.039)	(0.048)	(0.037)
Inc 0-10K	0.064	0.021	0.096	-0.112	0.010	-0.117
	(0.116)	(0.085)	(0.147)	(0.106)	(0.146)	(0.108)
Inc 10-20K	-0.056	0.086	0.131	0.042	-0.098	-0.057
	(0.092)	(0.059)	(0.099)	(0.077)	(0.099)	(0.072)
Inc 20-30K	-0.063	-0.004	0.238**	0.104	-0.056	-0.059
	(0.083)	(0.055)	(0.094)	(0.075)	(0.094)	(0.074)
Inc 30-40K	-0.082	0.010	0.029	-0.035	-0.035	-0.094
	(0.084)	(0.050)	(0.097)	(0.078)	(0.093)	(0.069)
Inc 40-50K	-0.042	-0.014	0.098	-0.071	-0.059	-0.149**
	(0.099)	(0.050)	(0.110)	(0.077)	(0.102)	(0.075)
Inc 50-60K	0.078	0.119**	0.076	0.013	0.033	0.008
	(0.105)	(0.057)	(0.107)	(0.081)	(0.109)	(0.079)
Inc 60-80K	0.019	0.023	0.182*	-0.040	-0.128	-0.119*
	(0.084)	(0.047)	(0.095)	(0.069)	(0.094)	(0.065)

Inc 80-100K	-0.111	-0.027	0.173	-0.087	0.023	-0.214***
	(0.092)	(0.051)	(0.108)	(0.087)	(0.120)	(0.081)
Inc 100-125K	0.110	0.130*	0.012	-0.077	0.145	-0.059
	(0.115)	(0.071)	(0.116)	(0.097)	(0.129)	(0.095)
Inc 125-150K	0.097	0.086	0.346***	0.165	0.042	-0.052
	(0.130)	(0.083)	(0.116)	(0.102)	(0.142)	(0.110)
Inc 150-200K	-0.011	0.032	0.040	0.046	-0.026	0.023
	(0.119)	(0.074)	(0.140)	(0.096)	(0.141)	(0.098)
Edu Degree	-0.214***	-0.101***	0.025	0.030	-0.015	-0.020
	(0.045)	(0.029)	(0.056)	(0.042)	(0.057)	(0.042)
SAH Excell	-0.144	-0.274***	0.165	0.031	0.064	-0.060
	(0.109)	(0.068)	(0.118)	(0.092)	(0.106)	(0.089)
SAH Vgood	-0.227**	-0.205***	0.205**	0.156**	0.134	-0.046
	(0.093)	(0.066)	(0.100)	(0.076)	(0.086)	(0.076)
SAH good	-0.160*	-0.187***	0.112	0.078	0.159*	-0.006
	(0.091)	(0.065)	(0.095)	(0.073)	(0.082)	(0.073)
SAH Fair	-0.023	-0.093	0.127	0.125*	0.171*	0.016
	(0.097)	(0.070)	(0.096)	(0.074)	(0.088)	(0.073)
PHI	0.059	0.048	0.233**	0.115*	0.129	-0.054
	(0.080)	(0.040)	(0.097)	(0.069)	(0.092)	(0.061)
PAHI	-0.058	-0.013	0.303***	0.374***	0.209**	0.198***
	(0.065)	(0.044)	(0.079)	(0.063)	(0.088)	(0.068)
PHI both	0.092*	0.022	0.267***	0.263***	0.247***	0.166***
	(0.050)	(0.032)	(0.053)	(0.042)	(0.055)	(0.041)
Risk: Avg.	-0.159	-0.003	0.095	0.087	0.089	-0.071
	(0.198)	(0.081)	(0.196)	(0.171)	(0.150)	(0.157)
Risk: Ab. avg.	-0.129	0.021	0.119	0.121	0.008	0.020
	(0.195)	(0.078)	(0.189)	(0.167)	(0.142)	(0.152)
Risk: Substantial	-0.201	-0.027	-0.047	-0.021	0.011	-0.078
	(0.195)	(0.080)	(0.189)	(0.166)	(0.141)	(0.151)
Constant	1.329***	0.648***	0.887*	0.244	0.100	0.137
	(0.448)	(0.244)	(0.460)	(0.330)	(0.450)	(0.370)
Observations	482	482	482	482	482	482
R^2	0.131	0.152	0.198	0.259	0.160	0.184
Hausman χ^2 (P-val)	(0.035)		(0.122)		(0.279)	

Note: See Table A3.

Table A6: Linear regressions on use and expectations: Childless singles only

	Physiotherapist		Naturopath		Massage	
	Past	Expected	Past	Expected	Past	Expected

Age	-0.008	0.004	-0.009	0.007	-0.005	0.006
	(0.013)	(0.010)	(0.008)	(0.006)	(0.012)	(0.010)
Age^2	0.000	-0.000	0.000	-0.000	0.000	-0.000
	(0.000)	(0.000)	(0.000)	(0.000)	(0.000)	(0.000)
Male	-0.099**	-0.111***	-0.027	-0.043***	-0.148***	-0.113***
	(0.043)	(0.031)	(0.019)	(0.016)	(0.036)	(0.029)
Employed	0.042	0.007	-0.014	-0.011	0.018	0.042
	(0.044)	(0.034)	(0.019)	(0.018)	(0.036)	(0.031)
NSW	0.109	0.075	-0.047	0.023	-0.056	-0.051
	(0.100)	(0.098)	(0.090)	(0.046)	(0.120)	(0.122)
VIC	0.084	0.041	-0.013	0.026	-0.055	-0.066
	(0.102)	(0.099)	(0.093)	(0.047)	(0.123)	(0.124)
QLD	0.165	0.113	-0.008	0.019	0.014	-0.023
	(0.104)	(0.100)	(0.091)	(0.046)	(0.126)	(0.125)
SA	0.195	0.098	-0.038	0.010	-0.094	-0.116
	(0.119)	(0.111)	(0.095)	(0.049)	(0.127)	(0.127)
WA	0.099	0.075	-0.023	0.001	-0.075	-0.047
	(0.117)	(0.107)	(0.093)	(0.048)	(0.128)	(0.131)
TAS	0.087	0.081	-0.054	-0.020	0.040	-0.080
	(0.154)	(0.133)	(0.091)	(0.050)	(0.165)	(0.136)
NT	-0.140	-0.050	-0.112	-0.029	-0.274	-0.184
	(0.240)	(0.157)	(0.120)	(0.074)	(0.201)	(0.141)
City	0.032	0.066**	0.036**	0.016	0.088***	0.063**
	(0.044)	(0.031)	(0.015)	(0.018)	(0.033)	(0.027)
Inc 0-10K	-0.100	-0.082	0.035	-0.021	-0.047	-0.111**
	(0.080)	(0.067)	(0.035)	(0.026)	(0.064)	(0.053)
Inc 10-20K	0.058	0.032	0.036	0.045	0.007	-0.010
	(0.083)	(0.059)	(0.032)	(0.029)	(0.067)	(0.056)
Inc 20-30K	0.011	0.030	0.030	0.026	0.001	0.020
	(0.078)	(0.057)	(0.030)	(0.027)	(0.067)	(0.057)
Inc 30-40K	0.072	0.018	-0.003	-0.012	0.018	-0.028
	(0.084)	(0.059)	(0.025)	(0.025)	(0.073)	(0.061)
Inc 40-50K	0.053	-0.050	0.085*	0.012	0.134	-0.001
	(0.090)	(0.060)	(0.047)	(0.035)	(0.085)	(0.064)
Inc 50-60K	0.168*	0.125	0.118**	0.054	0.115	0.035
	(0.098)	(0.077)	(0.053)	(0.038)	(0.088)	(0.071)
Inc 60-80K	0.017	-0.039	0.048	-0.006	-0.043	-0.106*
	(0.077)	(0.052)	(0.034)	(0.025)	(0.068)	(0.056)
Inc 80-100K	0.145	0.073	0.053	0.050	0.048	-0.063
	(0.105)	(0.078)	(0.052)	(0.050)	(0.101)	(0.071)
Inc 100-125K	-0.064	0.005	0.044	0.076	-0.094	-0.054
	(0.103)	(0.080)	(0.067)	(0.062)	(0.085)	(0.082)
Inc 125-150K	-0.051	0.016	0.035	0.102	-0.079	-0.025

	(0.114)	(0.095)	(0.068)	(0.073)	(0.084)	(0.086)
Inc 150-200K	-0.033	-0.013	-0.026	0.005	-0.045	-0.014
	(0.122)	(0.092)	(0.030)	(0.038)	(0.103)	(0.100)
Edu Degree	-0.110**	-0.055	-0.007	-0.033	-0.087**	-0.067*
	(0.052)	(0.039)	(0.027)	(0.022)	(0.043)	(0.036)
SAH Excell	0.049	-0.019	0.098*	0.018	0.147	0.007
	(0.113)	(0.083)	(0.055)	(0.046)	(0.090)	(0.070)
SAH Vgood	-0.110	-0.022	0.046**	0.024	0.031	0.037
	(0.090)	(0.063)	(0.023)	(0.026)	(0.066)	(0.052)
SAH good	-0.115	-0.058	0.026	-0.009	0.013	0.001
	(0.087)	(0.061)	(0.018)	(0.025)	(0.062)	(0.050)
SAH Fair	-0.074	0.009	0.026	0.011	0.023	0.014
	(0.093)	(0.066)	(0.021)	(0.027)	(0.066)	(0.051)
PHI	0.153*	0.034	0.089**	0.029	0.182**	0.101*
	(0.083)	(0.053)	(0.044)	(0.024)	(0.077)	(0.057)
PAHI	0.197**	0.233***	-0.026*	0.002	0.128*	0.126**
	(0.077)	(0.064)	(0.014)	(0.021)	(0.067)	(0.060)
PHI both	0.226***	0.167***	0.036*	0.048***	0.090**	0.084***
	(0.048)	(0.036)	(0.019)	(0.018)	(0.040)	(0.031)
Risk: Avg.	0.144	0.067	-0.003	-0.007	0.035	0.067
	(0.141)	(0.101)	(0.118)	(0.090)	(0.142)	(0.073)
Risk: Ab. avg.	0.085	0.079	-0.060	-0.005	0.010	0.116*
	(0.135)	(0.097)	(0.116)	(0.089)	(0.137)	(0.067)
Risk: Substantial	0.062	0.037	-0.064	-0.015	-0.024	0.060
	(0.136)	(0.097)	(0.115)	(0.090)	(0.136)	(0.068)
Constant	0.159	-0.038	0.255	-0.059	0.241	0.025
	(0.346)	(0.266)	(0.217)	(0.165)	(0.314)	(0.266)
Observations	482	482	482	482	482	482
R^2	0.151	0.167	0.128	0.098	0.162	0.139
Hausman χ^2 (P-val)	(0.067)		(0.018)		(0.007)	

Note: See Table A3.

Figure A1: Coefficient estimates childless singles – Hospital and dentist use

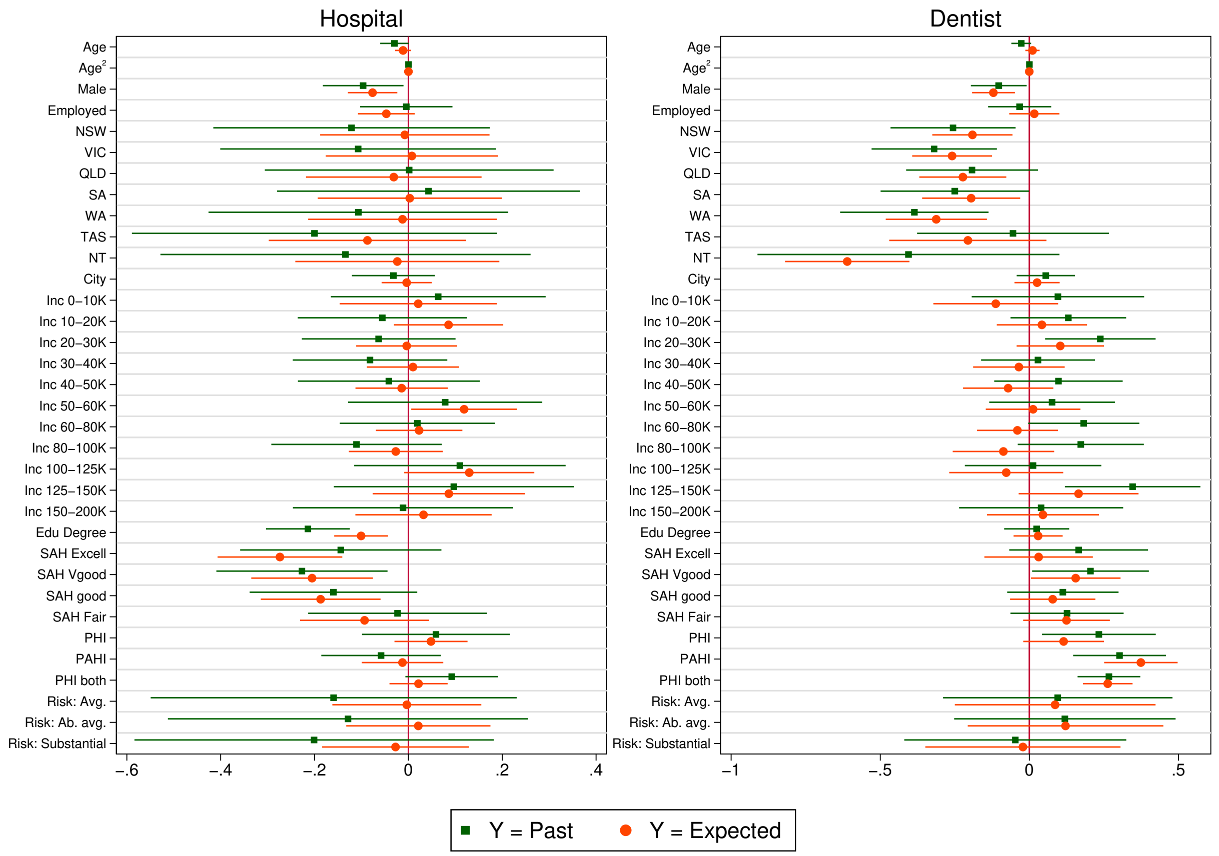

Note: Displayed are coefficient estimates and 95% confidence intervals (robust standard errors) from linear regression on an indicator for actual health service use in the last 12 months (squares) and expected probability of health service use in the next 12 months (circles). n=1,528.

Figure A2: Coefficient estimates childless singles – Optometrist and physiotherapist use

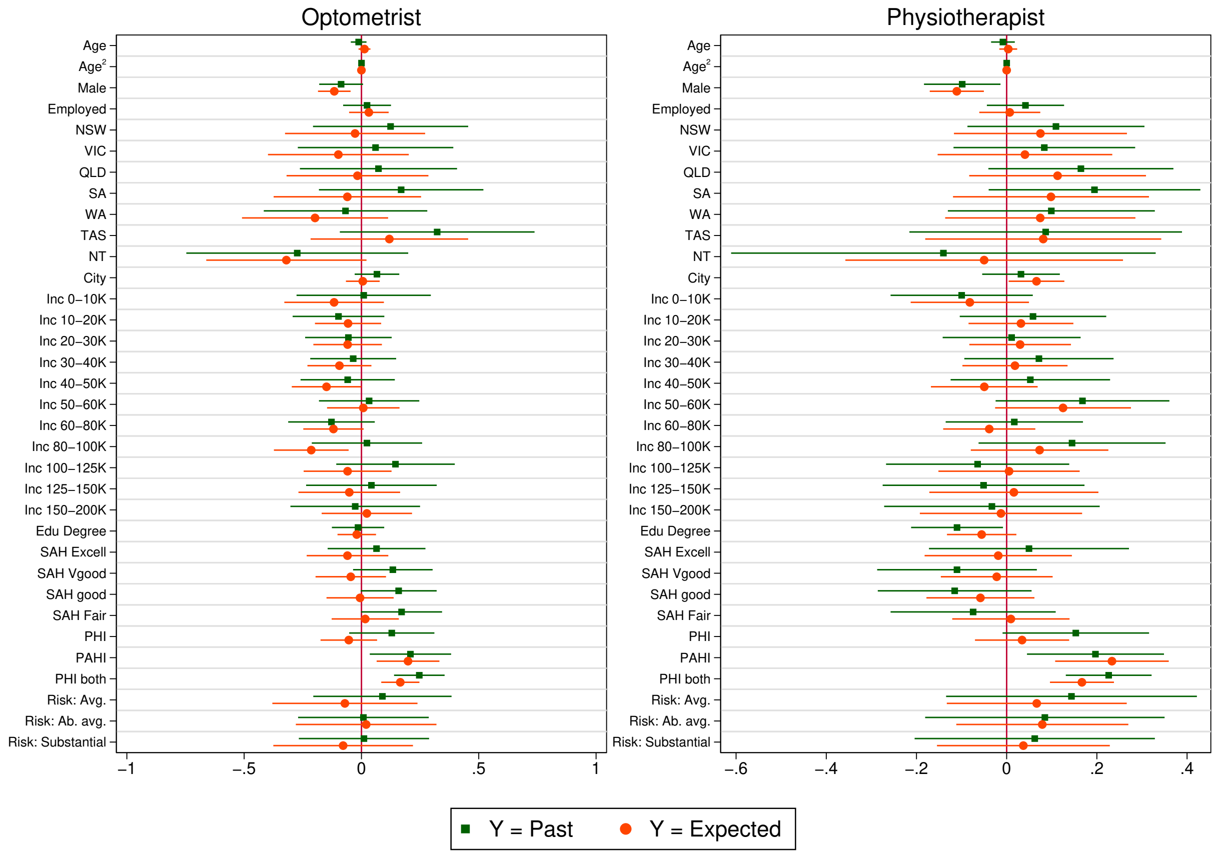

Note: See Figure A1

Figure A3: Coefficient estimates childless singles – Naturopath and massage therapist use

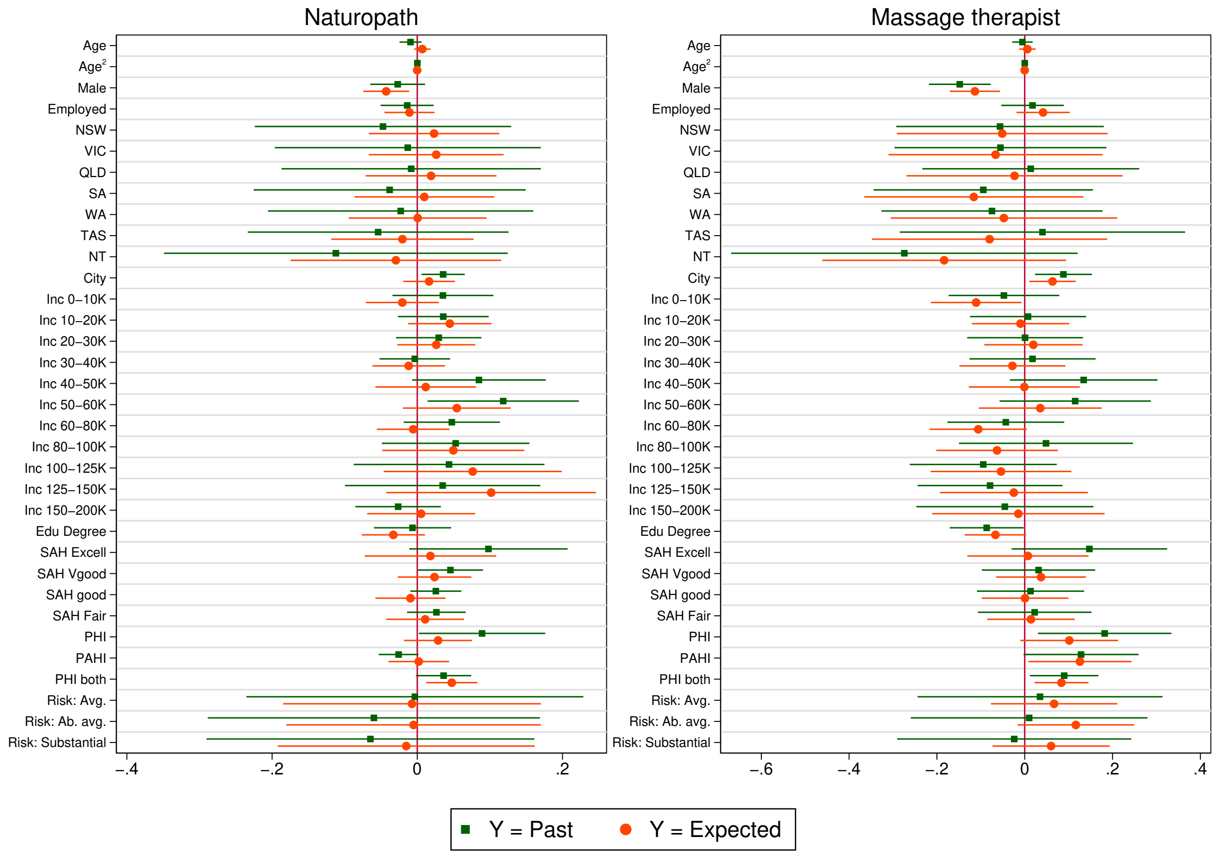

Note: See Figure A1

Figure A4: ROC curves

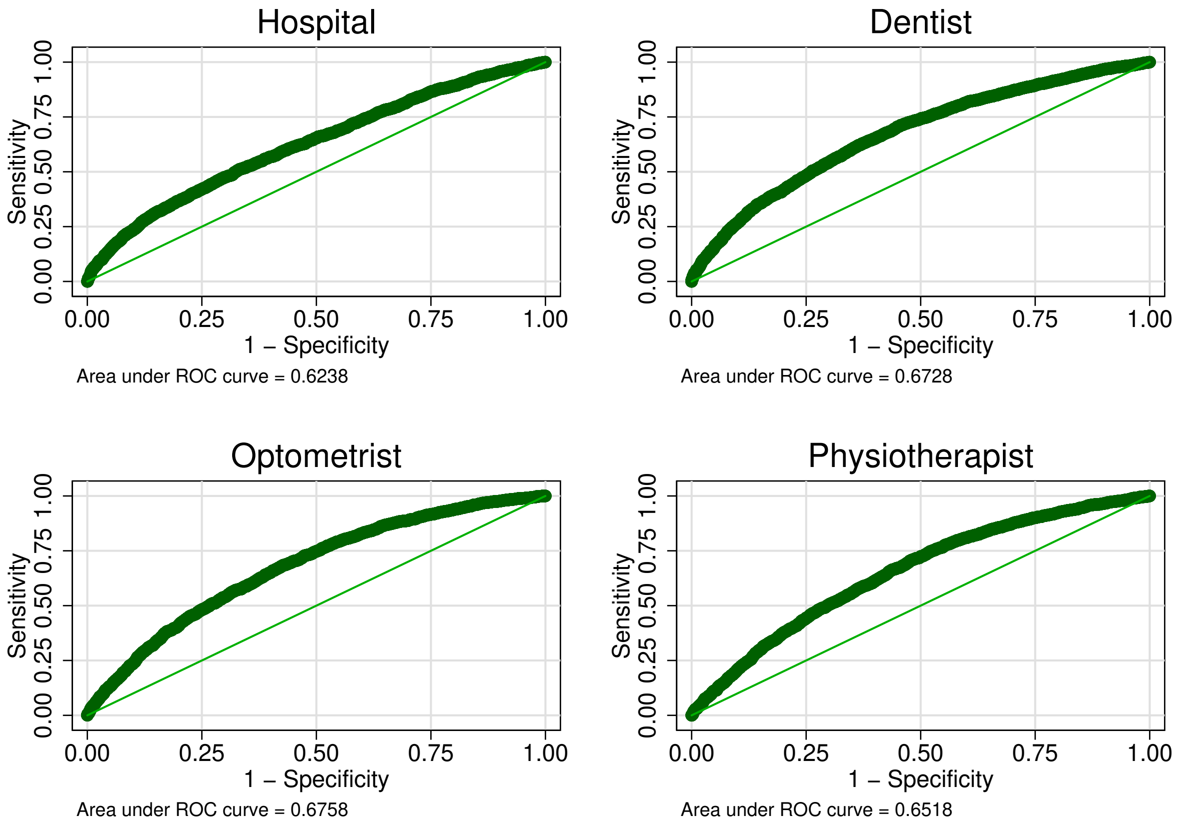

Note: The ROC curves are based on lasso logit estimates using the 2013 wave of HILDA. The tuning parameter was selected using K-fold cross validation and the preferred subset of covariates chosen based on a lowest deviance criterion. Sensitivity is the fraction of correctly identified positive outcome cases; specificity is one minus the fraction of correctly identified negative outcome cases. The area between the ROC curve and the 45 degree line (the ROC curve in a model with no predictive power) gives a measure of model fit ranging from 0 to 1, with higher values indicating better fit.

B Comparison between health service utilization variables in Online Survey and HILDA

As discussed in Section 2, because people are asked about health service visits generally in the Online Survey, it is possible that some of the variation in responses (to both subjective expectations and health service use in the last 12 months) is on behalf of other persons (e.g. spouses, children). To explore this, I compare the frequencies of reported health service utilization between the Online Survey and HILDA. Table B1 reports the raw differences and the differences after adjusting for covariates using propensity score matching.

Table B1: Mean utilization in Online Survey and HILDA

	Mean		Difference	
	Online sample	HILDA	Raw	Conditional
Hospital	0.317	0.270	0.048***	0.057***
			(0.012)	(0.021)
N	1,528	10,610	12,138	10,833
Hospital	0.317	0.204	0.113***	0.124***
(no emergency)			(0.011)	(0.020)
N	1,528	10,609	12,137	10,832
Dentist	0.573	0.532	0.042***	0.036*
			(0.014)	(0.019)
N	1,528	11,096	12,624	10,832
Optometrist	0.477	0.263	0.214***	0.212***
			(0.012)	(0.019)
N	1,528	10,612	12,140	10,834
Physiotherapist	0.270	0.252	0.018	0.015
			(0.012)	(0.019)
N	1,528	11,096	12,624	10,890

Note: Conditional differences are based on propensity score estimates using a logit model to predict selection into the experimental sample. Controls are the variables in Table A1. Robust Abadie-Imbens standard errors in parentheses. * $p < 0.10$, ** $p < 0.05$, *** $p < 0.01$.

Reported frequencies for hospitalizations and optometrist visits are higher in the Online Survey, which is consistent with responses being partially on behalf of others. However, visits to dentists are only significant at 10% after conditioning on observables; differences for physiotherapist are small and insignificant. In HILDA, people are specifically told not to include emergency care as part of self-reported hospital admissions (they are then asked about emergency visits separately). There is no such restriction in the Online Survey, and the smaller gap when emergency care is included in the HILDA hospitalization variable indicates that people did include emergency visits in their reported hospital use in the Online Survey.

If people are reporting on behalf of others, this is most likely to manifest in families, with people reporting on behalf of spouses and children. This suggests focussing on singles

if we want measures of expectations and prior utilization that reflect personal care only. To explore this I repeat the comparisons in Table B1 for singles only (see Table B2).

Table B2: Mean utilization in Online Survey and HILDA: Singles only

	Mean		Difference	
	Online sample	HILDA	Raw	Conditional
Hospital	0.256	0.276	-0.021	-0.018
			(0.022)	(0.028)
N	1,528	2,143	2,625	2,246
Hospital	0.256	0.191	0.064***	0.073***
(no emergency)			(0.020)	(0.027)
N	482	2,142	2,624	2,245
Dentist	0.527	0.469	0.058**	-0.023
			(0.025)	(0.029)
N	482	2,383	2,865	2,253
Optometrist	0.383	0.254	0.129***	0.123***
			(0.022)	(0.031)
N	482	2,144	2,626	2,247
Physiotherapist	0.226	0.212	0.014	0.009
			(0.020)	(0.028)
N	482	2,383	2,865	2,253

Note: See Table B1

After restricting attention to singles, differences between samples shrink, and only the difference for optometrist remains significant after matching. People may understate their visits to optometrists in HILDA because they are asked whether they visited an optometrist about their *health* (see Table A2). A common reason people visit optometrists in Australia is to purchase new corrective eyewear, which can be a purely cosmetic choice. People may have ignored these visits when responding to the HILDA survey. In practice, it is not clear whether we should want people to include or exclude visits that do not involve a clinical consultation. From the perspective of private health insurance, the main cost incurred by insurers are replacement of corrective eyewear (e.g. glasses and frames). Insurers can incur these costs regardless of whether there is a clinical consultation.

Overall, the results indicate that if we restrict the sample to singles, responses in the Online Survey are likely to predominately reflect own expected and actual health service use.
